# 2360 Engaging, capturing, and integrating the voice of the customer and collaborator in a clinical and translational science program

**DOI:** 10.1017/cts.2018.252

**Published:** 2018-11-21

**Authors:** Boris Volkov, Jennifer Cieslak, Brook Matthiesen

**Affiliations:** CTSI, University of Minnesota

## Abstract

OBJECTIVES/SPECIFIC AIMS: This presentation will highlight the framework, domains, and approaches of the “Engaging the Voice of the CTS Customer and Collaborator System” created at the University of Minnesota Clinical and Translational Science Institute (CTSI) in response to the need to improve the stakeholder engagement, quality, efficiency, consistency, and transparency of the clinical and translational work. This system addresses 3 important results-based accountability measures/questions: “What should we do?”, “How well did we do it?”, and “Is anyone better off?”. According to Woolf (2008), “translational research means different things to different people.” Social networks and systems that support translational processes and outcomes are complex, nonlinear, and multidisciplinary (Smith *et al.*, 2017). In this highly uncertain and fluid context, the input of program stakeholders is paramount to move translation forward. NCATS Strategic Plan (2016) directs the grantees to engage patients, community members and nonprofit organizations meaningfully in translational science and all aspects of translational research. Engagement of stakeholders throughout the lifecycle of a translational research project ensures the project processes and outcomes are relevant to and directly address their needs and will be more readily adopted by the community. “Customer” (among other terms are Beneficiary, Collaborator, Client, Community, Consumer, Service User, etc.) is a person, organization, or entity who directly benefits from service delivery or program (Friedman, 2005). Customers can be: direct and indirect, primary and secondary, internal and external. Our analysis of CTS stakeholders (“Who are our customers/collaborators?”) produced the following list of customers and collaborators: researchers, University departments, translational science workforce, patients, community members and entities, nonprofit organizations, industry collaborators, NCATS/NIH, CTSA hub partners, and CTSI staff. The “Voice of the Customer” (VOC) is the term used to describe the stated and unstated needs or requirements of the program’s customer. The “voice of the customer” is a process used to capture the feedback from the customer (internal or external) to provide the customers with the best quality of service, support, and/or product. This process is about being proactive and constantly innovative to capture the changing needs of the customers with time. Related to the VOC is the concept of user innovation that refers to innovations developed by consumers and end users. Experience shows that sometimes the best product or a process concept idea comes from a customer (Yang, 2007: p. 20). Capturing and utilizing such ideas are also relevant to VOC and can be operationalized and implemented as a valuable strategy. The University of Minnesota CTSI’s key objectives, goals, and uses of engaging the VOC and collaborator are as follows: (1) Engage CTSA customers (“relevant stakeholders”) in multiple aspects of translational science and look for opportunities to include their perspective (per NCATS strategic principles). (2) Inform continuous improvement, strategic management, and M&E efforts, the identification of customer needs and wants, comprehensive problem definition and ideation, new concept development and optimization. (3) Synergize NCATS and partner expectations and campus/hub needs. (4) Translate VOC into functional and measurable service requirements. METHODS/STUDY POPULATION: A case study of the programmatic and methodological approach/technique development. The VOC at the UMN CTSI has been captured in a variety of ways: regular and ad hoc surveys, interviews, focus groups, Engagement Studios, formal call for patient/community ideas and proposals, informal conversations, customer/community membership and participation in the Advisory Boards and Executive Leadership Team meetings, and observations. Our VOC variables and metrics assess customer needs, wants, knowledge, and skills; customer satisfaction with processes and outcomes; and customer ideas for improvement and innovation. The ensuing customer feedback and other data have been used to identify and incorporate the important attributes needed in the CTSI processes, products, and dissemination. UMN CTSI partners in engaging and capturing the VOC include our past, current, and potential customers and collaborators, communities, program staff and service providers, program administration, communication staff, M&E team, internal and external data collectors. RESULTS/ANTICIPATED RESULTS: The proposed comprehensive approach shows sound promise to enhance customer and collaborator engagement, critical thinking, learning, strategic management, evaluation capacity and improvement within clinical and translational science organizations. DISCUSSION/SIGNIFICANCE OF IMPACT: This structured approach’s impact is significant in that it fills the current gap in the practice, literature, and methodology and offers a practical example of a “practice that works” for CTR (and other) organizations and programs striving to improve their stakeholder engagement and program impact. Leveraging and synergizing the VOC and community engagement approaches can help CTS organizations advance beyond capturing individual project/service experiences to drawing a holistic portrait of an institution-level (and, potentially, a nation-level) translational science program.

**References**

**Friedman M**. *Trying Hard Is Not Good Enough: How to Produce Measurable Improvements for Customers and Communities*. Trafford, 2005.

**National Center for Advancing Translational Sciences**. *NCATS Strategic Plan* [Internet], 2016. NIH (https://ncats.nih.gov/strategicplan)

**Smith C,** ***et al***. Toward a science of translational science. *Journal of Clinical and Translational Science* 2017; **1**: 253–255.

**Woolf SH**. The meaning of translational research and why it matters. *JAMA* 2008; **29**: 211–213.

**Yang, K**. *Voice of the Customer Capture and Analysis*. US: McGraw-Hill Professional, 2007.

